# Unique metabolic traits of *Pseudomonas bharatica* CSV86^T^: a promising candidate for biodegradation and up-cycling of aromatics

**DOI:** 10.1128/jb.00445-25

**Published:** 2026-01-14

**Authors:** Prashant S. Phale, Tushar Dhamale, Satyajit Subhash, Sandesh E. Papade, Omkar P. Ingale, Braja Kishor Saha, Sravanti T. Kasarlawar

**Affiliations:** 1 Department of Biosciences and Bioengineering, Indian Institute of Technology-Bombay 29491, Powai, Mumbai, India; Geisel School of Medicine at Dartmouth, Hanover, New Hampshire, USA

**Keywords:** *Pseudomonas bharatica *CSV86^T^, aromatic metabolism, preferential utilization of aromatics, plant growth promoting activity, metabolic engineering

## Abstract

*Pseudomonas* spp. are ubiquitous, metabolically versatile bacteria that exhibit remarkable adaptability to thrive in diverse ecological niches contaminated with aromatics, such as pesticide-polluted agricultural soils, industrial wastewater, and lignin-based waste. This review highlights the unique genetic and metabolic traits of *Pseudomonas bharatica* CSV86^T^, a novel soil bacterium capable of degrading a wide range of aromatics, including lignin-derived phenylpropanoid compounds. Unlike other pseudomonads, aromatic metabolism in strain CSV86^T^ is not subjected to carbon catabolite repression by simple carbon sources. Instead, it preferentially utilizes aromatics over glucose/glycerol and co-metabolizes them with organic acids, circumventing a major bottleneck in biodegradation. The strain is plasmid-free and naphthalene metabolism pathway genes are present on conjugatively transferable integrative conjugative element, ICE*nah*CSV86, offering potential for genetic bio-augmentation. Notably, the strain grows slowly on glucose and metabolizes it exclusively *via* phosphorylative pathway, as oxidative routes are absent. Beyond aromatic metabolism, it displays multifarious plant growth-promoting and beneficial eco-physiological traits for niche colonization and adaptation, crucial for restoration of contaminated sites. Collectively, these unique traits position strain CSV86^T^ as a niche-adapted alternative to model biodegradation strains, such as *P. putida* KT2440. Its potential can be further leveraged through metabolic engineering for detection, degradation, and up-cycling of aromatic pollutants.

## INTRODUCTION

**Fig 1:**
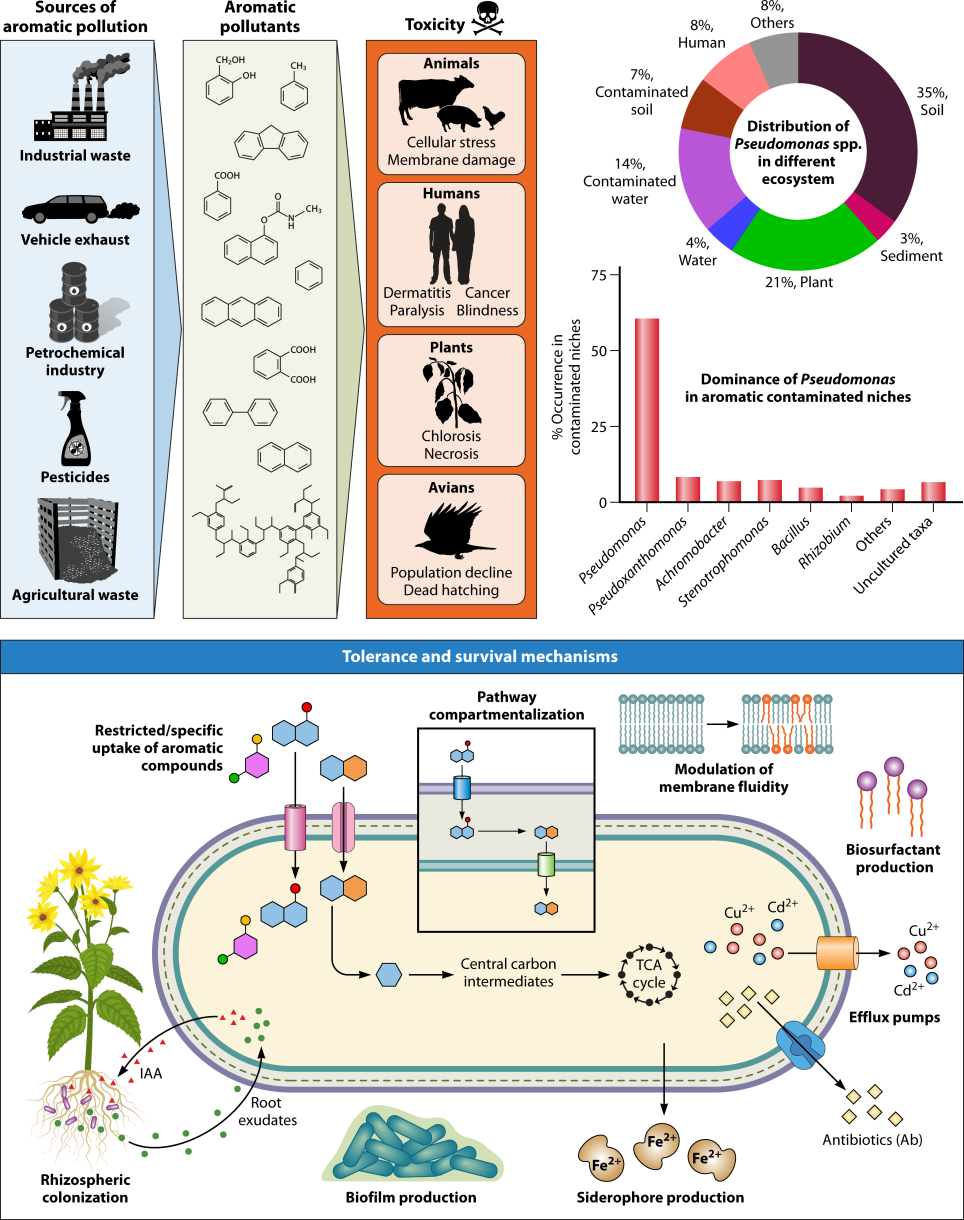
Source and toxicity of aromatic compounds, abundance of pseudomonads in aromatic-contaminated niches, and strategies employed for tolerance and survival.

Aromatic compounds, ranging from simple mono-aromatics like benzene and toluene to complex polycyclic aromatics and their substituted derivatives, are among the most prevalent and persistent pollutants in the environment. They originate from a wide range of natural as well as anthropogenic sources, including industrial effluents, vehicle exhaust, plastics, pesticides, burning of crop residues, etc. (1, 2). Natural processes, such as degradation of lignin from plant biomass, contribute significantly to the environmental load of aromatic compounds. Due to their highly reduced and resonance-stabilized ring structures, aromatics are chemically stable and hydrophobic, making them recalcitrant to degradation. This leads to their accumulation in soils, sediments, surface, and groundwater, thereby polluting various ecological compartments (3, 4). Many of these aromatic pollutants are reported to be mutagens, endocrine disruptors, carcinogens and eco-toxicants, posing serious risks to not only humans but the entire biota. Thus, removal of these pollutants is essential to safeguard ecosystem function and public health (Fig. 1; [5]).

Compared with available remediation strategies, biodegradation, which leverages natural capacity of microbes to detoxify and/or degrade pollutants, has emerged as one of the most sustainable, cost-effective, and environmentally friendly approaches. Among various microbes, pseudomonads dominate diverse aromatic-contaminated niches and are most versatile in terms of their adaptability towards multiple ecosystems (Fig. 1; [6]). Several metagenomic and culture-dependent studies have reported the predominance of pseudomonads in contaminated sites, reinforcing their ecological significance in biodegradation (6–10). To thrive in such niches, pseudomonads employ an array of strategies for tolerance and degradation of aromatics. These include the use of efflux pumps, modulation of membrane fluidity, chemotaxis toward pollutants, biosurfactant production, specific transporters for aromatic uptake, pathway compartmentalization to reduce cytoplasmic toxicity, secretion of siderophores for iron acquisition, and reverse carbon catabolite repression, among various other strategies (Fig. 1). Their genome is highly plastic and can acquire new genes for tolerance and degradation *via* horizontal gene transfer. Additionally, the ability for rhizospheric colonization and various eco-physiological traits including plant growth promotion and biocontrol potential makes them attractive candidates for bioremediation as well as ecosystem restoration (6).

This review highlights the unique metabolic repertoire of *Pseudomonas bharatica* CSV86^T^ for efficient biodegradation of toxic aromatics, niche colonization, adaptation, and plant growth promotion. It discusses the engineering of strain CSV86^T^ with genetic elements from *Pseudomonas* sp. C5pp to address persistent challenges in biodegradation, such as limited metabolic diversity, carbon catabolite repression, metabolic crosstalk, and intermediate toxicity. Further, comparison of strain CSV86^T^ with the model strain *P. putida* KT2440 underscores its potential as an alternative host within the genus *Pseudomonas* for metabolic engineering aimed at degradation and up-cycling of aromatic pollutants.

## *PSEUDOMONAS BHARATICA* CSV86^T^: A NOVEL SPECIES WITH UNIQUE PREFERENCE FOR AROMATIC METABOLISM

**Fig 2:**
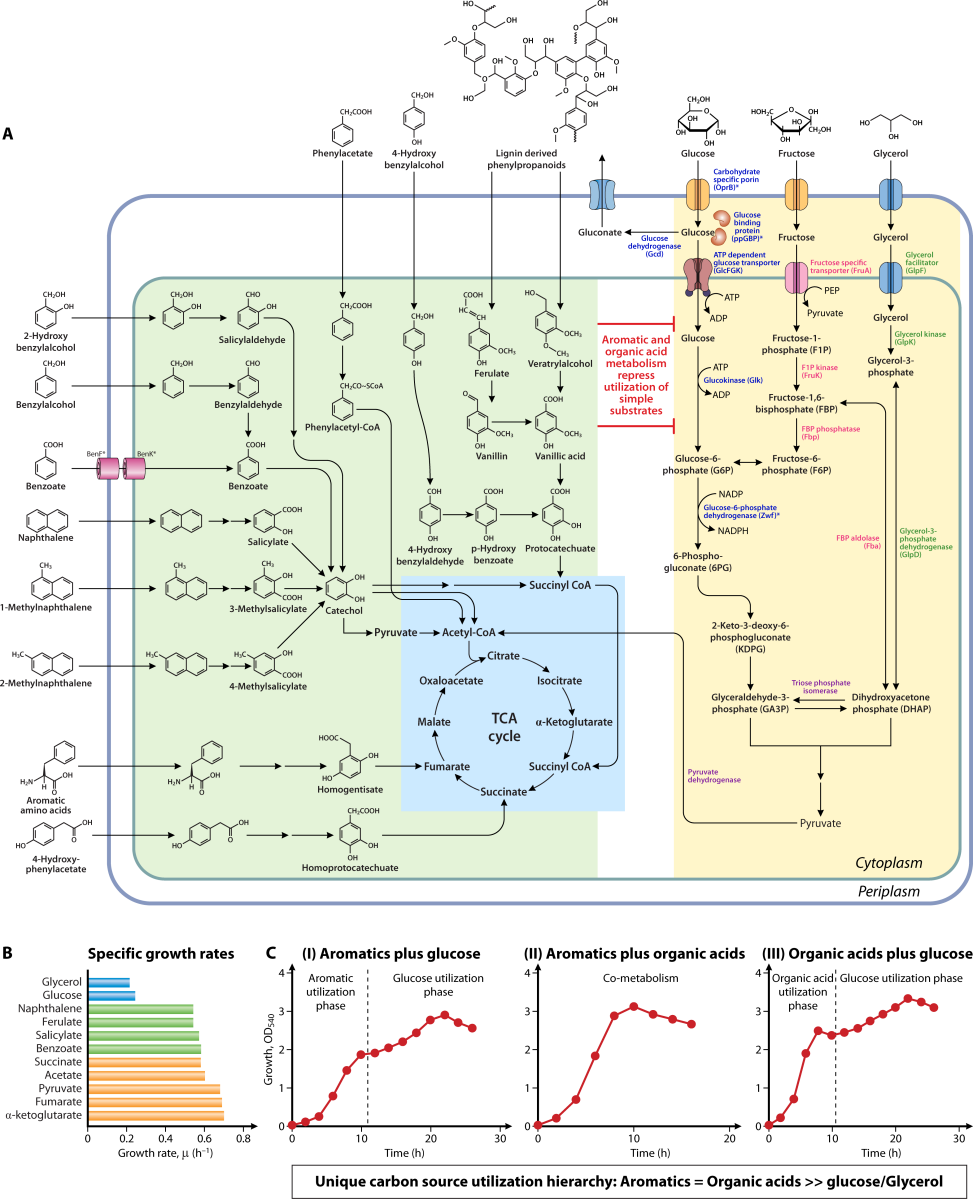
Aromatic metabolism and carbon source utilization hierarchy of *Pseudomonas bharatica* CSV86^T^. (**A**) Metabolic pathways for aromatics and organic acids are depicted in green and blue panels, respectively. Metabolism of non-preferred carbon compounds is depicted in the yellow panel. Proteins involved in the uptake and utilization of glucose, fructose, and glycerol are shown in dark blue, red, and dark green, respectively. Experimentally characterized proteins are marked with an asterisk. (**B**) Bar graph depicts the specific growth rates of strain CSV86^T^ on various carbon compounds: sugar and sugar alcohol (blue), aromatics (green), and organic acids (orange). (**C**) Growth profile of strain CSV86^T^ on a mixture of (I) aromatics plus glucose, (II) aromatics plus organic acids, and (III) organic acids plus glucose. Dotted lines are used to separate the two log phases.

*Pseudomonas bharatica* CSV86^T^ was isolated from petroleum-contaminated soil (Bengaluru, India) as a naphthalene degrader. It also utilizes a wide range of aromatic compounds, such as methylnaphthalenes, benzyl alcohol, hydroxybenzyl alcohols, benzaldehyde, benzoate, salicylate, *p*-hydroxybenzoate, phenylacetate, *p*-hydroxyphenylacetate, as well as lignin-derived phenylpropanoids like veratryl alcohol, veratraldehyde, veratric acid, ferulic acid, vanillate, and vanillin as the sole source of carbon and energy (Fig. 2, Table 1; [11, 12]). Strain CSV86^T^ rapidly utilizes organic acids like acetate, citrate, succinate, α-ketoglutarate, fumarate, as well as malate. It metabolizes glucose, fructose, and glycerol but failed to utilize galactose, arabinose, mannose, maltose, gluconate, and 2-ketogluconate as carbon sources (Fig. 2; [12–15]). The unique feature of this strain is to preferentially utilize aromatics over glucose and co-metabolize aromatics with organic acids (6, 16).

**TABLE 1:** Comparison of genetic, metabolic, and eco-physiological traits of *Pseudomonas bharatica* CSV86^T^ and *Pseudomonas putida* KT2440

Feature		*Pseudomonas bharatica* CSV86^T^	*Pseudomonas putida* KT2440
Habitat		Isolated as naphthalene degrader from petroleum product contaminated soil in India (11)	Plasmid cured derivative of *P. putida* mt-2 that was isolated from garden soil in Japan (17)
Safety level		Lacks major pathogenicity and virulence factors (risk group-1) (6)	HV1 certified by FDA (18)
Genome features	Genome properties	6.79 Mb with 62.72 G+C mol%, plasmid free (6)	6.18 Mb with 61.5 G+C mol%, plasmid free (19)
	Genomic islands and mobile genetic elements	18 genomic islands with three transposons, 11 IS elements, and 3 integrative conjugative elements (ICE*nah*CSV86, PBGI-1, and PBGI-2) (6)	61 genomic islands containing 4 prophages, 2 transposons, and 36 IS elements (20, 21)
		Ability for conjugative transfer of ICE*nah*CSV86 to *Stenotrophomonas maltophila* CSV89 (22, 23)	No ICE integrative conjugative elements present
			No report of KT2440 as conjugal donor
Aromatic metabolism	Native degradation pathways present for	Naphthalene, 1-/2-methylnaphthalenes, benzylalcohol, 2-/4-hydroxybenzylalcohol, benzaldehyde, benzoate, salicylate, *p*-hydroxybenzoate, phenylacetate, *p*-hydroxyphenylacetate, veratryl alcohol, veratraldehyde, veratric acid, ferulic acid, vanillate, and vanillin (6, 24)	Benzoate, 4-hydroxybenzoate, coniferyl alcohol, *p*-coumarate, ferulate, caffeate, vanillate, vanillin, nicotinate, gallate, benzylamine, phenylacetate, phenylethylamine, phenylhexanoate, phenylheptanoate, phenyloctanoate (25, 26)
	When equipped with natural plasmids from other pseudomonads	Not determined	Degrades toluene, xylene, 3-methylbenzoate when equipped with TOL plasmid pWW0 from *P. putida* mt-2 (27) Degrades naphthalene when equipped with NAH7 catabolic plasmid from *P. putida* G7 (28) Degrades biphenyl and salicylate when equipped with *bph-sal* element (conjugative transposon) from *P. putida* KF715 (29)
	Engineered pathways	Carbaryl (30)	Fenitrothion and permethrin (31) Chlorpyrifos and carbofuran (32), Methyl parathion, fenpropathrin, cypermethrin, Carbaryl (33) Dinitrotoluene (34). Bis(2-hydroxyethyl) terephthalate (35), syringate (36)
	Central aromatic intermediates/routes	Catechol, protocatechuate, homo-protocatechuate, and homo-gentisate (24)	Catechol, protocatechuate, and homo-gentisate (26)
		Gentisate route is absent (6)	Homo-protocatechuate and gentisate routes are absent (26)
Carbon source utilization hierarchy		Aromatic metabolism is not repressed by glucose or organic acids (14)	Aromatic metabolism is repressed by glucose (37)
		All aromatics are preferred over glucose in Fe-replete conditions (6, 14)	Benzoate and toluene are co-metabolized with glucose in iron-replete conditions (38, 39)
		Iron limitation does not alter the preferential utilization of aromatics over glucose	Benzoate is preferred over glucose only in Fe-limited conditions (38)
		Aromatics are co-metabolized with organic acids (14)	Not reported
Glucose metabolism	Growth on MSM supplemented with glucose (0.25% w/v)	Prolonged lag phase (~10 h), low growth rate (0.23 h⁻¹), low biomass (final OD_540_ = 1.2) (15)	Short lag phase (~2h), high growth rate (0.54 h^−1^), high biomass (final OD_540_ = 3.5)
	Primary pathways	Glucose is metabolized exclusively *via* phosphorylative pathway (13, 15)	Glucose is metabolized *via* phosphorylative as well as oxidative routes (40)
		Genes encoding oxidative pathway enzymes are absent or frame-shifted except *gcd* gene, which encodes a functional glucose dehydrogenase (15)	~90% of glucose is converted to gluconate and fluxed through oxidative routes while 10% metabolized *via* phosphorylative pathway (40)
	Catalytic efficiency of ZwfA	Lower (*K*_cat_/*K*_m_= 1.7 for NADP^+^) (15)	Higher (*K*_cat_/*K*_m_= 7.3 for NADP^+^) (41)
	Effect of aromatics/organic acids	Glucose metabolism repressed by organic acids and aromatics (14)	Glucose metabolism is repressed by organic acids (42, 43)
Environmental adaptations	Heavy metal resistance	Co, Zn, Cd, Cu (24)	Co, Zn, Cd, Ni, Pb, (21)
	Antibiotic resistance	Resistant to ampicillin, piperacillin, ticarcillin, and cephalosporin (24)	Resistant to chloramphenicol, sulfamethoxazole, trimethoprim, rifampicin, and cefoxitin (44)
	Salinity tolerance	5.3% NaCl (w/v) (45)	4% NaCl (w/v) (46)
	Fusaric acid resistance	Yes (45)	Yes (47)
	Production of indole acetic acid	Yes (45)	No (48)
	Solubilization of inorganic phosphate	Yes (45)	Yes (49)
	Production of siderophores	Yes (45)	Yes (48)
	Plant growth promotion	Demonstrated with wheat, mung bean, and fenugreek crops (45)	Demonstrated with maize, soybean, and *Arabidopsis* (48)

Strain CSV86^T^ was initially identified as *Pseudomonas putida* based on its morphological and biochemical properties (11). With the advent of genome sequencing, the growing availability of *Pseudomonas* genomes enabled genome‐resolved taxonomic analyses, revealing that members of the *P. putida* G-group are phylogenetically diverse. Moreover, unique metabolic features of strain CSV86^T^ distinguished it physiologically from other strains of *P. putida*, thus warranting proper taxonomic classification. On the basis of 16S rRNA gene phylogeny, strain CSV86^T^ appeared closest to *Pseudomonas japonica* WLT. However, lower genetic indices, differences in biochemical traits (organic acid and amino acid assimilation), and fatty acid composition suggested taxonomic distinctiveness of strain CSV86^T^. Thus, polyphasic taxonomy combined with detailed taxono-genomic analysis supported demarcation of strain CSV86^T^ as a novel species. It was named as *Pseudomonas bharatica* CSV86^T^, after Bharat, i.e., Sanskrit name for India (12, 24). The strain CSV86^T^ (=MTCC 2445^T^ =ICMP 24314^T^ =JCM 34937^T^) is the type strain of *Pseudomonas bharatica* sp. nov.

## METABOLIC DIVERSITY FOR AROMATIC DEGRADATION

The aromatic degradation property of strain CSV86^T^ is chromosomally encoded and involves “upper pathways” that generate metabolic intermediates (such as catechol, protocatechuate, homoprotocatechuate, and homogentisate) that are channeled into “lower pathways” and eventually enter the TCA cycle, reflecting metabolic diversity (Fig. 2A; [11, 50–52]). In strain CSV86^T^, naphthalene is metabolized to salicylate by a series of enzymatic steps catalyzed by several oxygenases and dehydrogenases. The oxidative decarboxylation of salicylate yields catechol, which undergoes *meta*-ring cleavage and is funneled to the TCA cycle (Fig. 2A; [11]). Enzymes for metabolism of naphthalene to salicylate (upper pathway) are encoded by *nah* operon (*nahAaAbAcAdBFCED*), while those for conversion of salicylate to TCA cycle intermediates (lower pathway) are encoded by *sal* operon (*nahGTHINLOMKJX*). These operons are co-transcribed and induced by salicylate-bound NahR, a LysR type transcriptional regulator encoded by gene *nahR* present upstream to *sal* operon (22, 52). The metabolism of 1- and 2-methyl naphthalene follows a similar route as that described for naphthalene. However, strain CSV86^T^ also employs a “detoxification pathway” where naphthalene dioxygenase converts methylnaphthalenes to hydroxymethyl naphthalenes, which are then oxidized due to promiscuous activities of benzylalcohol dehydrogenase (BADH) and benzaldehyde dehydrogenase (BZDH) to naphthoic acid, that occurs as a dead-end product (11, 50, 51). The specific role of BADH and BZDH in strain CSV86^T^ is to metabolize benzyl alcohol to benzoate *via* benzaldehyde, 2-hydroxybenzylalcohol to 2-hydroxybenzoate (salicylate) *via* 2-hydroxybenzaldehyde, and 4-hydroxybenzylalcohol to 4-hydroxybenzoate *via* 4-hydroxybenzaldehyde (Fig. 2A; [50]). These wide substrate range aromatic dehydrogenase and aromatic aldehyde dehydrogenase are encoded by genes present in a cluster with AraC family transcriptional regulator (52).

Strain CSV86^T^ metabolizes benzoate to catechol that is ring cleaved at the *ortho* position and funneled to the TCA cycle *via β*-ketoadipate route (Fig. 2A). Gene clusters *benABCDKcatAbenEF* encode enzymes for uptake and metabolism of benzoate to *cis,cis*-muconate, while *cat*BC encode enzymes for conversion of *cis,cis*-muconate to *β*-ketoadipate pathway intermediates. These clusters are regulated by BenR and CatR, with benzoate and *cis,cis*-muconate as inducers, respectively (52–54). In contrast, 2-hydroxybenzoate (salicylate) is metabolized *via* catechol *meta*-cleavage route, encoded by *sal* operon as described earlier (Fig. 2A). 4-Hydroxybenzoate is hydroxylated by 4-hydroxybenzoate 3-monooxygenase (encoded by *pobA*) to yield protocatechuate which is further metabolized through *ortho*-cleavage route. The genes for protocatechuate metabolism are present in a cluster as *pcaRKFTBDCJHG* (Fig. 2A; [50, 52]). Catechol generated from naphthalene, methylnaphthalene, 2-hydroxybenzylalcohol, and salicylate exclusively undergoes *meta*-cleavage, while catechol from benzylalcohol and benzoate degradation route is fluxed through *ortho*-cleavage route. This mutually exclusive substrate-specific aromatic degradation network is observed in few *Pseudomonas* species capable of utilizing naphthalenes, benzyl alcohols, as well as benzoate and highlights their metabolic versatility and plasticity.

Phenylacetate is metabolized *via* phenylacetyl-CoA by action of phenylacetyl-CoA ligase, and pathway enzymes are encoded by gene clusters *paaABCDEF* and *paaGHIJKLMN* (52, 55). On the other hand, 4-hydroxyphenylacetate is metabolized *via* homoprotocatechuate *ortho* cleavage route, and metabolic enzymes are encoded by gene cluster *hpaAGEDFHI* (52, 56). Homogentisate (a common intermediate in amino acid metabolism) is metabolized *via ortho* ring-cleavage route, and pathway enzymes are encoded by gene cluster *hmgABC* (52).

Strain CSV86^T^ degrades lignin sulfonate (OD_540nm_ = 0.35) over a period of 4–5 days. However, it metabolizes lignin pathway intermediates, such as veratryl alcohol, veratraldehyde, ferulate, vanillin, and vanillate (OD_540nm_ = 1–1.5) through the phenylpropanoid pathway in 10 to 14 h (57). Veratryl alcohol is sequentially converted to veratraldehyde and then to veratric acid, which is demethylated to yield vanillate. Whereas, ferulate is converted to vanillate *via* vanillin. Vanillate is further hydroxylated to protocatechuate, which enters the *ortho* ring cleavage route and fluxes into the TCA cycle. The genes encoding these phenylpropanoids pathways are present in three clusters, i.e., *fer* locus (*ech-vdh-fcs-kct-acdh*), *ver* locus (*verBA-tdh-vanK-oprD*), and *van* locus (*vanAB*), that are induced by ferulate, veratric acid, and vanillate, respectively (52, 57).

## GLUCOSE METABOLISM

In pseudomonads, glucose is metabolized *via* a cyclic network involving the Entner-Doudoroff (ED), Embden-Meyerhof-Parnas (EMP), and Pentose Phosphate (PP) pathways (40). First, glucose passes through the outer membrane *via* OprB porin and reaches the periplasm where it can be fluxed through three convergent routes: one intracellular phosphorylative route and two peripheral oxidative routes to form 6-phosphogluconate (6PG), which is further funneled into central carbon metabolism (58, 59). In the phosphorylative pathway, glucose is transported into the cytoplasm and phosphorylated by glucokinase (Glk) to yield glucose-6-phosphate (G6P), which is then converted to 6PG by the action of glucose-6-phosphate dehydrogenase (Zwf) and 6-phosphogluconolactonase. Alternatively, in oxidative pathways, glucose in the periplasm is oxidized to gluconate and 2-ketogluconate (2-KG) by glucose dehydrogenase (Gcd) and gluconate 2-dehydrogenase (Gad), respectively. Gluconate and 2-KG are transported into the cytoplasm and converted to 6PG. This intermediate is converted to 2-keto-3-deoxy-6-phosphogluconate (KDPG), which is then split to yield pyruvate and glyceraldehyde-3-phosphate that enter the central carbon metabolism (40, 58, 60). In many *Pseudomonas* strains, such as *Pseudomonas putida* KT2440, these three routes co-exist and function simultaneously with 90% of glucose fluxed through the peripheral oxidative routes and only a minor fraction (10%) fluxed through the intracellular phosphorylative route (Table 1). However, under glucose- or oxygen-limiting conditions, strains like *Pseudomonas aeruginosa* rely exclusively on the phosphorylative pathway and completely shut off oxidative routes (40, 61, 62).

In strain CSV86^T^, genes for glucose uptake and metabolism *via* intracellular phosphorylative pathway are present in three clusters: *gbp-glcFGK-oprB, zwfA-pgl-eda,* and *edd-glk-gltRII-hkR*, whereas genes responsible for oxidative pathway are either absent or have frameshift mutations. Metabolic analysis (whole-cell oxygen uptake, enzyme activity) and inability of CSV86Δ*gbp* to metabolize glucose suggested that strain CSV86^T^ lacks oxidative pathways, and glucose is metabolized exclusively *via* the intracellular phosphorylative route (Fig. 2A; [13, 63]).

**Fig 3:**
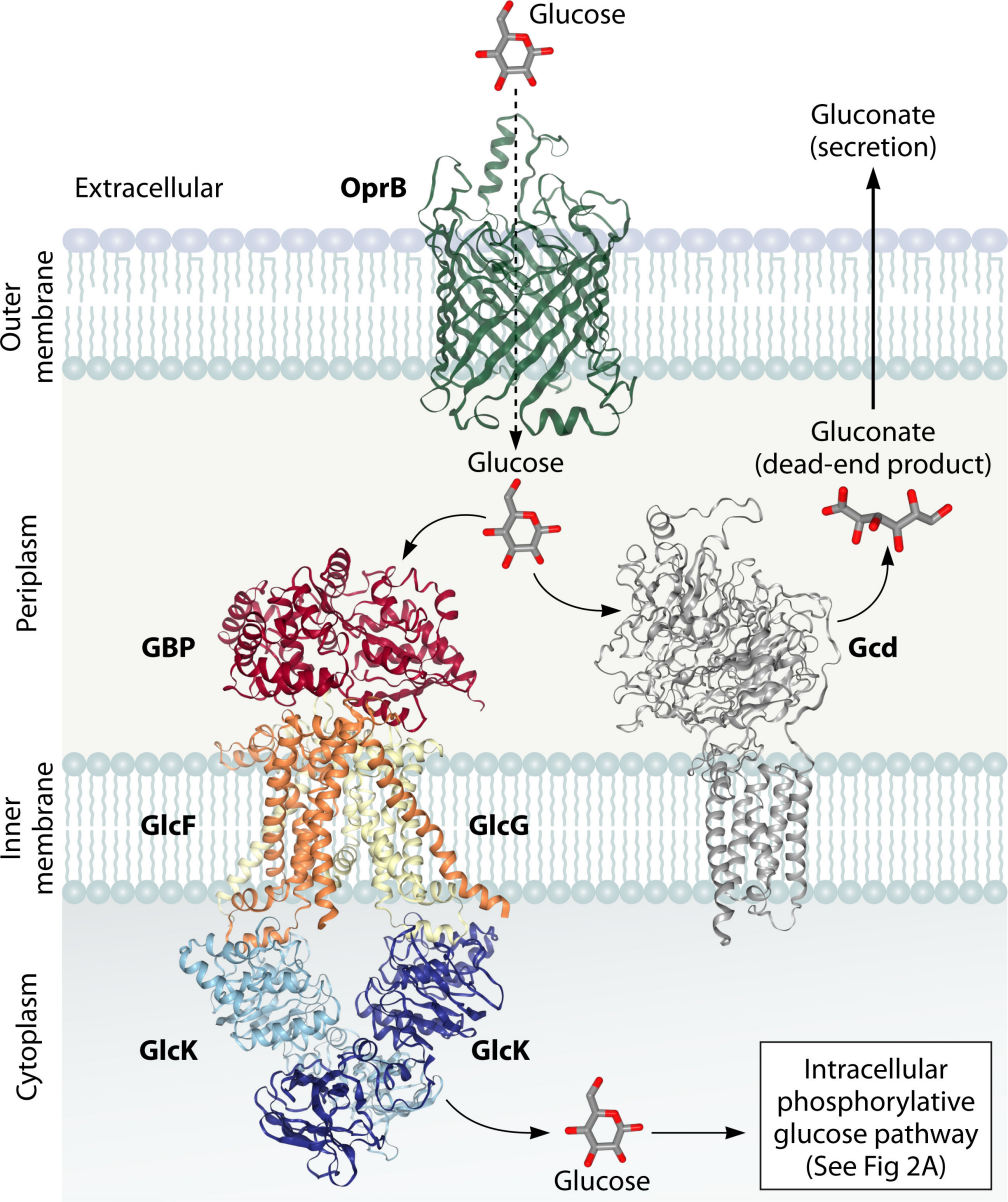
Glucose ABC-transport system in *Pseudomonas bharatica* CSV86^T^. OprB, outer membrane glucose protein; GBP, periplasmic glucose-binding protein; GlcF and GlcG, inner membrane glucose permeases; GlcK, glucose kinase, Gcd, periplasmic glucose dehydrogenase. 3D structure of GBP was retrieved from PDB (id 5DVF [67]), while other proteins were modeled using Alphafold3.

Glucose transport in strain CSV86^T^ is inducible, and genome analysis suggested the presence of an outer membrane protein OprB and a periplasmic glucose-binding protein (GBP)-dependent ABC-transporter (Fig. 3). The identification of the glucose-binding pocket in GBP was achieved through a combination of homology modeling, molecular docking, and site-specific mutagenesis studies, providing insights into substrate specificity and the structural basis of glucose recognition. Homology modeling of GBP revealed that it belonged to class II family of periplasmic binding proteins. The model showed the highest structural similarity to the GBP of *Thermus thermophilus* (ttGBP, RMSD: 0.64 Å). Molecular docking studies revealed six key residues (W35, W36, E41, K92, K339, and H379) as probable glucose-binding residues. Alanine substitution of these residues using site-directed mutagenesis resulted in a significant reduction in ^14^C-glucose-binding activity, validating their role in glucose binding *via* hydrogen bond formation. The substrate specificity of the purified protein was assessed by performing substrate displacement of GBP bound to ^14^C-glucose in the presence of 100-fold molar excess of various unlabeled compounds. Unlabeled glucose completely displaced ^14^C-glucose binding to GBP while other related monosaccharides, organic acids, and aromatics failed to display any significant displacement of ^14^C-glucose binding to GBP, suggesting that the binding pocket is very specific and can only accommodate glucose (64, 65). The high-resolution crystal structures of GBP in both unbound and sugar-bound forms provided important insights into the substrate specificity. GBP has a high affinity of 0.3 µM for glucose (1.25 Å) and lower affinity with ligands like galactose (1.8 Å) due to fewer hydrogen bonds. Despite its specificity for monosaccharides, GBP retains a structural fold characteristic of oligosaccharide-binding proteins. Structural adaptations in the binding pocket restrict it to accommodate only glucose, preventing binding of other structurally similar sugars (66).

Another notable feature is the presence of an intact *gcd* gene encoding glucose dehydrogenase (Gcd) that converts glucose into gluconate (dead-end product), which cannot be metabolized further due to the lack of oxidative pathways (Fig. 3). This might be the reason for low biomass of strain CSV86^T^ on glucose (OD_540_= ~1.2) compared with strain KT2440 (OD_540_= ~3.5) (Table 1). Despite its metabolic cost, we hypothesize that strain CSV86^T^ may benefit from gluconate secretion as it leads to reduction in the pH. This may confer ecological benefits like (i) negatively affecting the growth of other bacteria, thereby reducing competition and (ii) solubilization of inorganic minerals like iron, phosphate, potassium from their oxide forms which may promote bacterial as well as plant growth (38, 68).

Glucose-6-phosphate dehydrogenase (Zwf) is one of the key enzymes in the sole route for glucose metabolism in strain CSV86^T^. Similar to other *Pseudomonas* species, strain CSV86^T^ harbors three isozymes: ZwfA, ZwfB, and ZwfC, among which ZwfA plays a predominant role in glucose metabolism by catalyzing the conversion of G6P to 6-phosphogluconolactone with the concomitant generation of NADPH. However, the catalytic efficiency of ZwfA from strain CSV86^T^ (for its cofactor NADP^+^) is significantly lower (*K*_cat_/*K*_m_= 1.7) as compared with other pseudomonads like strain KT2440 (*K*_cat_/*K*_m_= 7.3) (Table 1; [15, 41]). This reduced efficiency might act as a metabolic bottleneck, reducing NADPH production and also slowing the rate of formation of intermediates like KDPG that is crucial for relieving HexR mediated repression of glucose metabolic genes. As a result, essential anabolic reactions and redox homeostasis may be impaired, leading to a prolonged lag phase and reduced specific growth rate on glucose. Overall, in strain CSV86^T^, the less efficient ZwfA, combined with the presence of a functional Gcd (which locks much of glucose as gluconate) and absence of peripheral oxidative routes, may collectively account for poor growth of strain CSV86^T^ on glucose (Table 1).

## UNIQUE CARBON SOURCE UTILIZATION HIERARCHY

In complex habitats, bacteria metabolize a variety of carbon sources but in a hierarchical manner, prioritizing easily utilizable and energy-rich substrates like glucose over complex substrates, such as aromatics (42, 69). This poses a significant challenge in biodegradation, as the presence of simple carbon sources in contaminated environments represses the pollutant degradation pathways in bacteria introduced for clean-up efforts. In pseudomonads, organic acids repress both glucose and aromatic metabolism, and glucose represses aromatic metabolism, establishing a carbon source utilization hierarchy: organic acids > sugars > aromatics (42). This carbon source utilization hierarchy is not stringently followed by some *Pseudomonas* species. For example, *Pseudomonas putida* M2 co-metabolizes glucose with lignin-derived aromatics, such as *p*-coumarate, ferulate, and vanillate, but neither of the carbon sources is completely utilized. This arises due to reciprocal repression as glucose exerts a strong repressive effect on aromatic pathway enzymes, while aromatics repress key glycolytic enzymes, leading to co-utilization but incomplete degradation. Similar to other *Pseudomonas* spp., the levels of sRNAs CrcY and CrcZ correlate with CCR strength, and disruption of *crc* only partially alleviates incomplete substrate utilization, suggesting a role of additional, unidentified regulatory networks that contribute to the metabolism of lignocellulosic derivatives (70).

However, in strain CSV86^T^, the aromatic metabolism is not suppressed by simple carbon sources like glucose or organic acids. Instead, strain CSV86^T^ preferentially metabolizes aromatics over glucose and co-metabolizes aromatics with organic acids. The growth rate on aromatics plus organic acid (μ = 0.55-0.6 h^−1^) is similar to that observed on aromatics alone. Thus, carbon source utilization hierarchy in strain CSV86^T^ is aromatics = organic acids > sugars (Fig. 2B and C and Table 1; [6, 14, 16, 53, 71]). Transcriptional and biochemical analyses revealed that genes encoding enzymes for glucose transport and metabolism, i.e., carbohydrate-specific porin OprB, periplasmic GBP, and glucose-6-phosphate dehydrogenase (ZwfA) are induced in the presence of glucose (53). They are transcriptionally repressed during metabolism of aromatics and organic acids even in the presence of glucose. Moreover, aromatic metabolic genes are not repressed at the transcriptional or translation level by glucose or organic acid metabolism. 2D gel electrophoresis and LC-MS confirmed glucose-dependent induction of OprB and GBP and their repression in the presence of aromatics and organic acids. The expression and glucose binding activity of GBP was found to be selectively repressed, resulting in inhibition of glucose transport. This transcriptional modulation of aromatic and glucose metabolic genes allows strain CSV86^T^ to prioritize aromatics and organic acids over glucose, leading to preferential utilization of aromatics over glucose (53, 56, 72).

In *Pseudomonas* spp., iron availability intricately regulates carbon metabolism. For example, *P. putida* KT2440 reroutes carbon flux toward gluconeogenic substrates (e.g., benzoate, succinate) under Fe limitation, increasing siderophore production by threefold and enhancing Fe extraction from minerals by sixfold (73). Conversely, Fe-replete conditions suppress siderophore production and prioritize glycolytic carbon (glucose) utilization for growth. This Fe-dependent metabolic re-routing directly impacts the carbon source utilization hierarchy in strain KT2440. In Fe-replete conditions (i.e., 35μM), benzoate and glucose are co-metabolized. While in Fe-limited conditions (i.e 38nM), benzoate (i.e., a gluconeogenic) is preferred over glucose (i.e., glycolytic) (73). Contrastingly, strain CSV86^T^ metabolizes benzoate preferentially over glucose even in Fe-replete conditions (18 μM), suggesting no effect of iron on carbon source utilization hierarchy (Table 1, Fig. 2C; [(53]).

In *Pseudomonas* spp., carbon source utilization hierarchy is a direct implication of CCR, which is mediated by the CbrAB–Hfq–Crc system: the global regulator Crc (with Hfq) binds to catabolite activity (CA) motifs in mRNAs encoding enzymes for metabolism of deferred or secondary carbon sources and represses their translation. This repression is relieved by small RNAs (CrcX/Y/Z) whose expression levels are controlled by the two-component system CbrAB and expressed when preferred carbon source is exhausted (43, 74–77). The genome of strain CSV86^T^ harbors genes encoding Crc, Hfq, and sRNAs CrcZ and CrcY, but its unique substrate preference suggests that these regulators either operate differently or some additional inputs are important. Moreover, knockout of *crc* in strain CSV86^T^ has no effect on its unique carbon source utilization hierarchy (63). The role of alternative regulators like PtsN^Ntr^ or effectors (KDPG relieving HexR repression) in determining carbon source preference in strain CSV86^T^ cannot be overlooked.

In strain CSV86^T^, similar to glucose metabolism, glycerol utilization is characterized by a lengthy lag phase and low growth rate (Fig. 2B; [14]). *P. putida* KT2440 shows similar phenotype on glycerol due to tightly controlled, metabolite-responsive regulatory circuits (78–80). Transcriptional repressor (GlpR) blocks expression of uptake and catabolic genes until its inducer (glycerol-3-phosphate) reaches threshold levels to create a positive feedback and bistable (all-or-none) expression that delays pathway activation. This ensures tight control but causes prolonged lag phase on glycerol compared to organic acids or aromatics. Such regulation allows metabolic bet-hedging, i.e., giving bacteria time to scout for alternative carbon sources (e.g., aromatics or organic acids) to avoid competition. The identical genetic arrangement of glycerol operon in strain CSV86^T^ and strain KT2440 suggests that they likely share the same mechanism and might be one of the reasons for strain CSV86^T^ to preferentially metabolize aromatics over glycerol.

## GENOMIC PLASTICITY AND SCOPE FOR GENETIC BIOAUGMENTATION

Strain CSV86^T^ contains a large genome of 6.79 Mb with 62.7 mol % G + C content, of which nearly 19 % is mobile genetic elements. Notably, it harbors 18 genomic islands including three integrative conjugative elements (namely ICE*nah*CSV86, PBGI-1, and PBGI-2) of >50 kb, highlighting its adaptive potential and genomic plasticity. ICE*nah*CSV86 harbors the *nah-sal* cluster responsible for naphthalene degradation, ICE-2 (PBGI-1) carries heavy metal resistance genes responsible for the transformation and efflux of heavy metals, such as Cd, Zn, and Co, and ICE-3 (PBGI-2) is a mixed function element that carries genes for antibiotic resistance, motility and phage-associated proteins (Table 1; [6, 22]).

The naphthalene degradation genes are part of ICE*nah*CSV86 that contains tRNA^Gly^ gene and an integrase, indicating acquisition by horizontal gene transfer. This is distinct from many other *Pseudomonas* strains, where the *nah* operons are often plasmid-borne (e.g., NAH7 in *P. putida* G7) or found on integrative conjugative elements and they may lose the degradation phenotype more readily. The coding sequences of the *nah* genes are highly conserved (>90% identity) across *Pseudomonas* spp., reflecting a common evolutionary origin and frequent horizontal gene transfer. The NAH7 plasmid carries the best characterized naphthalene degradation pathway, split into an upper pathway (naphthalene to salicylate) and lower pathway (salicylate to catechol entering central metabolism). Similar operonic arrangement is found among other *Pseudomonas* species with variability arising from gene rearrangements and partial deletions (81). ICE*nah*CSV86 could be conjugatively transferred to *Stenotrophomonas maltophilia* CSV89 with low frequency, endowing trans-conjugants with ability to preferentially utilize naphthalene over glucose (23). These analyses not only reveal that strain CSV86^T^ has acquired these mobile genetic elements through horizontal gene transfer but also indicates that it can be used to transfer this stable property to other bacteria through conjugation, which makes this organism a promising choice for genetic bioaugmentation (22, 52).

## ECO-PHYSIOLOGICAL TRAITS AND *IN SITU* APPLICATION

**Fig 4:**
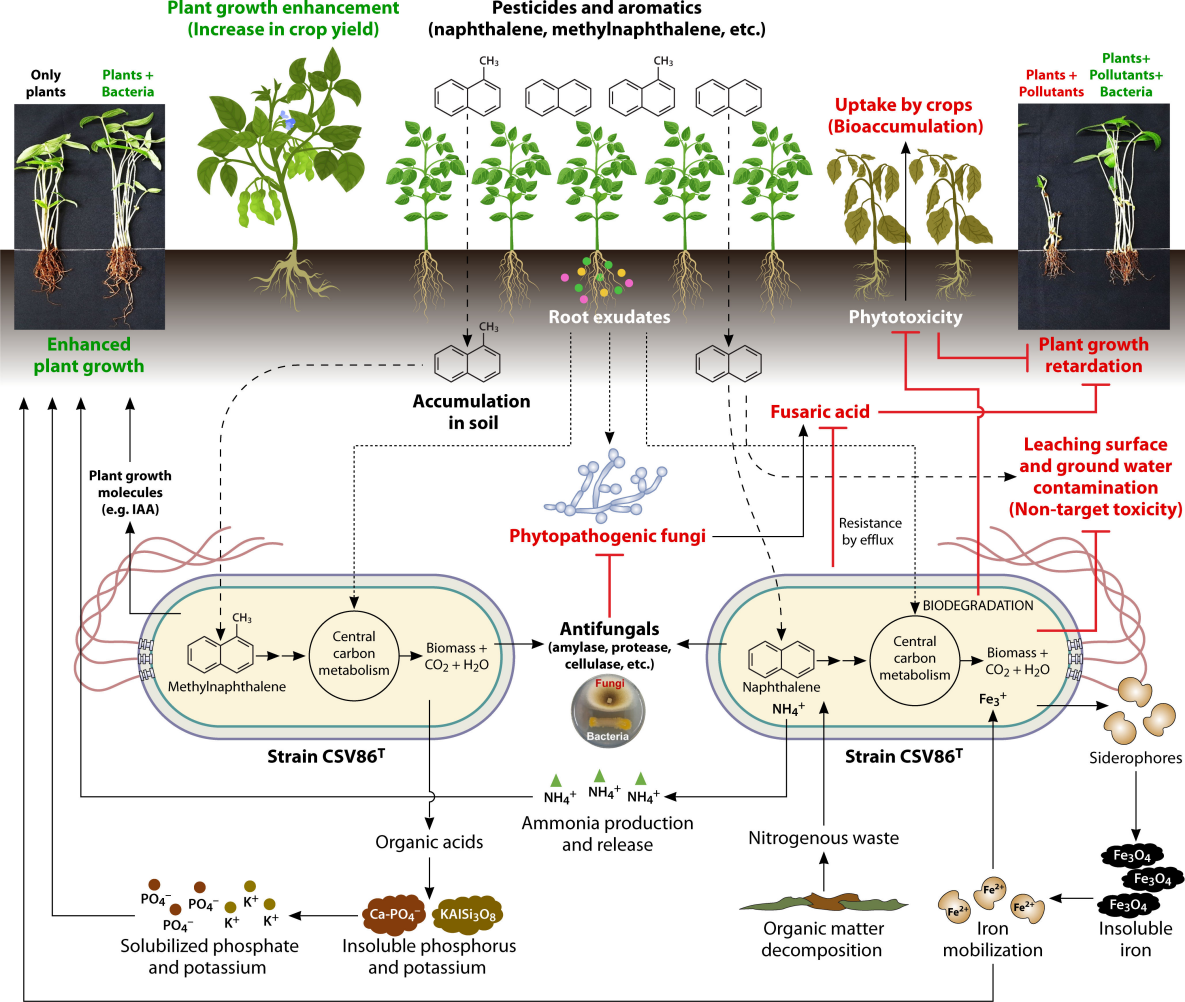
Plant growth-promoting, antifungal, and phytoprotective properties of *Pseudomonas bharatica* CSV86^ᵀ^. It possesses multifaceted plant growth-promoting traits like phosphate/potassium solubilization, IAA and ammonia production, siderophore-mediated iron mobilization as well as biocontrol traits, such as production of chitinase, protease against phytopathogenic fungi. It also degrades toxic agrochemicals, reducing toxicity to crop plants and non-target biota.

Application or introduction of bacteria for *in situ* clean-up of contaminated sites often faces challenges, such as competition with better-adapted native microflora for niche and nutrients, along with various abiotic stresses. *P. bharatica* CSV86^T^ is a promising soil bacteria that exhibit remarkable metabolic versatility for degradation of various aromatic pollutants. In addition to unique metabolic potential for aromatic degradation, it possesses several eco-physiological traits beneficial for *in situ*, niche adaptation, colonization, symbiosis with plants, stress tolerance, and survival under dynamic environmental conditions (Fig. 4).

Traits, such as biofilm formation, tolerance to biotic (e.g., fusaric acid, a mycotoxin) and abiotic (e.g., salinity and heavy metals) stressors permit survival in hostile environments and aid effective colonization on plant roots (82). Strain CSV86^T^ harbors multiple copies of *fusABCD* operon encoding a tripartite efflux system for the export of fusaric acid and shows resistance to high concentrations of fusaric acid (6, 45). It tolerates high salinity (up to 5%–6%) as well as heavy metals, such as Co²^+^, Zn²^+^, Cd²^+^, and Cu²^+^. It possesses genes for production of extracellular polysaccharide like alginate (*algAFJILXGEK448D*), adhesion and quorum sensing, which contribute to biofilm formation, a trait important for niche colonization and persistence (83).

Strain CSV86^T^ displays antifungal activity against phytopathogens, such as *Magnaporthe oryzae* and *Aspergillus* spp., leading to a significant reduction in seed infection rates compared with untreated controls in *in vitro* wheat germination assays (45) (Fig. 4). It exhibits amylase and cellulase activities that likely contribute to inhibition of fungal spore germination and hyphal growth (84, 85). Moreover, it exhibits several plant growth-promoting traits like solubilization of inorganic phosphorus (P) and potassium (K), likely *via* acid production (45, 86). It secretes indole-3-acetic acid (IAA), a plant growth hormone, *via* L-tryptophan-dependent pathways. Genes encoding amino acid ammonia-lyases and ammonium transporters support ammonia production from nitrogenous substrates, enhancing nutrient bioavailability. It possesses complete *pvdADEFHIJLMNOPQ* cluster for biosynthesis and uptake of mixed-type siderophores (pyoverdine) essential for iron acquisition under limiting conditions. Additionally, the presence of ACC deaminase activity suggests a role in modulating plant ethylene levels under stress, providing protection from both biotic and abiotic stressors (Fig. 4; [87]).

*In planta* application of strain CSV86^T^ (i.e., seed priming) significantly enhanced seed germination, growth and biomass of wheat, mung bean, and fenugreek. Its co-application with *Pseudomonas* sp. C5pp, *Pseudomonas* sp. PP4, and *Acinetobacter* sp. ISP4 (as consortium) showed the most robust effect on plant growth in *in soil* microcosm assays (Fig. 4; [45]). Its aromatic degradation capabilities also helped alleviate the phytotoxic effects of naphthalene in spiked soils, further supporting their promising potential in bioremediation and sustainable agriculture.

## *PSEUDOMONAS BHARATICA* CSV86^T^: A PROMISING HOST FOR METABOLIC ENGINEERING

Most natural bacteria employed for biodegradation face several challenges like abiotic stressors, poor bioavailability and toxicity of pollutants, nutrient limitation, competition with native flora, limited degradation pathways, carbon source utilization hierarchy, etc. limiting their effectiveness in environmental clean-up (42). With the advent of synthetic biology, these challenges can be addressed by metabolic engineering, which adopts a "patchwork" approach, i.e., genes and regulatory circuits from various bacteria are recombined into robust hosts/chassis to expand scope of degradation and valorization of pollutants (78, 88). *Pseudomonas putida* KT2440 has been the model chassis for biodegradation and synthetic biology owing to its genetic tractability, metabolic versatility, and extensive molecular toolbox (25, 89). However, distinctive genetic and metabolic features of *Pseudomonas bharatica* CSV86^T^, make it a promising, niche-adapted alternative host to strain KT2440 for metabolic engineering and synthetic biology applications. Strain CSV86^T^ has been used as host to demonstrate optimization of aromatic-inducible promoter systems, express and localize *Pseudomonas*-origin enzymes and engineering for preferential degradation of toxic pesticide Carbaryl (1-naphthyl-*N*-methylcarbamate; 30, 90); these aspects are described in detail in following sections.

## OPTIMIZATION OF AROMATIC COMPOUND–INDUCIBLE PROMOTERS IN CSV86^T^

The naphthalene degradation pathway enzymes in strain CSV86^T^ are encoded by *nah* and *sal* operons with P*nah* and P*sal* promoters, regulated by LysR-type transcriptional activator, NahR, which respond to salicylate as an inducer (22, 52, 81). The promoters (P*trc*/*lacI*, P*nah*, P*sal*, and P*sal*/*nahR*) were fused to a reporter gene *mcbC* (that encodes 1-naphthol 2-hydroxylase, 1NH) from *Pseudomonas* sp. C5pp (Carbaryl pesticide degrader [91]) and cloned into pSEVA234, a broad-host range vector. The reporter enzyme 1NH is a FAD-dependent group A monooxygenase, and it catalyzes hydroxylation of 1-naphthol using NAD(*P*)H as a cofactor that can be monitored spectrophotometrically, making it a sensitive and reliable reporter (90, 92, 93).

The P*trc*/*lac*I system showed tight IPTG-regulated expression in *E. coli* but exhibited leaky expression in both strains KT2440 and CSV86^T^, highlighting differences in activity of *lac* repressor in *E. coli* and pseudomonads. The P*nah* promoter was found to be constitutive in *E. coli*, even in the absence of inducer or NahR, suggesting it to be leaky, similar to that observed in native strain CSV86^T^. However, P*nah* showed no activity in strain KT2440, likely due to absence of NahR and/or CCR (22, 90). Unlike *E. coli*, where CCR acts at the transcriptional level, pseudomonads employ a post-transcriptional CCR mechanism mediated by the Crc-Hfq complex (77). The P*sal* promoter alone showed no activity in *E. coli* and strain KT2440, as these strains lack the NahR regulator, while significant activity was observed in CSV86^T^ as it harbors chromosomally encoded NahR. Moreover, when P*sal* was co-expressed with NahR (P*sal*/NahR), a strong, salicylate-inducible expression was achieved in all three hosts, suggesting NahR is essential for activation of P*sal* (22, 90). Among all promoters tested, P*nah* was found to be the best promoter in strain CSV86^T^. Importantly, the reporter 1NH activity was consistently higher in strain CSV86^T^ and KT2440 as compared with *E. coli*, indicating that *Pseudomonas* hosts offer a more favorable transcriptional, translational, and protein folding environment for expressing *Pseudomonas*-origin proteins.

## EXPRESSION AND PERIPLASMIC LOCALIZATION OF CARBARYL HYDROLASE IN CSV86^T^

**Fig 5:**
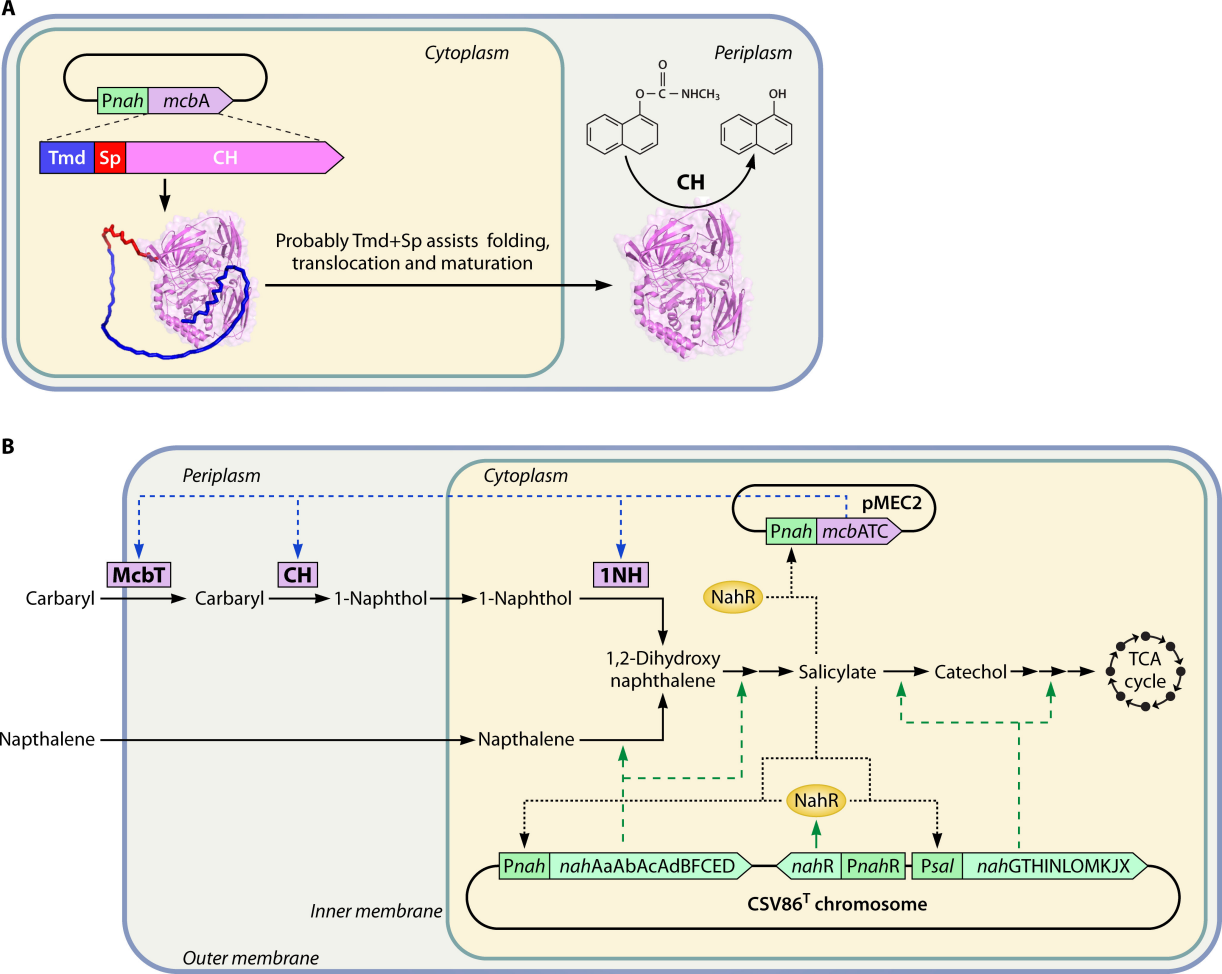
Integration of genetic and metabolic elements of *Pseudomonas bharatica* CSV86^T^ and *Pseudomonas* sp. C5pp. (**A**) Expression and periplasmic localization of Carbaryl hydrolase (CH) from strain C5pp in CSV86^T^. (**B**) Metabolic engineering of strain CSV86^T^ to degrade Carbaryl by expression of putative Carbaryl transporter McbT in the outer membrane, CH in the periplasm, and 1-naphthol 2-hydroxylase (1NH) in the cytoplasm from strain C5pp. Dashed lines (----) represent genes encoding respective degradation enzymes. Dotted lines (**^……^**) represent regulation of operons by NahR.

In *Pseudomonas* sp. C5pp, the Carbaryl degradation is initiated by periplasmic enzyme, Carbaryl hydrolase (CH), which hydrolyzes Carbaryl into 1-naphthol (91). CH contains a unique N-terminal region comprising a 96-amino-acid transmembrane domain (Tmd) plus signal peptide (Sp) referred to as Tmd+Sp that facilitates its translocation into the periplasm (94). During transport, the Tmd+Sp domain is cleaved, and the mature CH enzyme is localized within the periplasmic space. Functional studies on truncated versions of Tmd+Sp domains fused to either CH or GFP revealed that deletion of either the Tmd or Sp disrupted translocation, while deletion of both elements resulted in misfolding and aggregation of CH into inclusion bodies. This indicated that the Tmd+Sp domain is not only critical for periplasmic targeting but also for the correct folding and stability of CH in *E. coli* (94). Efforts to heterologously express CH with its Tmd+Sp domain in *E. coli* resulted in poor expression levels compared with native strain C5pp. These limitations were addressed by expressing the full-length gene encoding CH (with Tmd+Sp) under the P*nah* promoter in strain CSV86^T^ (90), in which CH effectively expressed and localized in the periplasmic space (Fig. 5A). The periplasmic extracts of CSV86^T^ exhibited a four-fold higher CH activity compared with that expressed in *E. coli*, indicating superior expression, folding, and maturation. Furthermore, CH purified from the periplasmic fraction of CSV86^T^ showed kinetic parameters similar to native CH purified from strain C5pp (90). This suggests probable utility of Tmd+Sp domain for translocation and proper folding of large proteins in the periplasm of strain CSV86^T^.

## ENGINEERING OF CSV86^T^ FOR CARBARYL DEGRADATION

The naphthalene degradation pathway in strain CSV86^T^ and the Carbaryl degradation pathway in strain C5pp share common metabolic steps, i.e., 1,2-dihydroxynaphthalene to salicylate (81). This metabolic convergence was exploited by introducing only the initial steps necessary to convert Carbaryl into 1,2-dihydroxynaphthalene and relying on the native downstream salicylate pathway of strain CSV86^T^ for further metabolism (Fig. 5B; [30]).

Genes encoding CH and 1NH from strain C5pp were expressed under the control of P*nah* (reported for its strong and leaky activity) in strain CSV86^T^ to yield the engineered strain CSV86-MEC1 (30). In CSV86-MEC1, CH was effectively localized into the periplasm where it hydrolyzed Carbaryl into 1-naphthol, which was then hydroxylated by cytoplasmic 1NH into 1,2-dihydroxynaphthalene. Native enzymes encoded by the *nah* and *sal* operon of strain CSV86^T^ subsequently converted 1,2-dihydroxynaphthalene into salicylate and funneled into TCA cycle *via* the catechol *meta*-cleavage route. However, the growth of CSV86-MEC1 on Carbaryl was slower compared to that of strain C5pp. Genome analysis of strain C5pp revealed the presence of a gene encoding a probable outer membrane transporter McbT, and *in silico* analysis predicted it to be involved in Carbaryl uptake. Thus, McbT was co-expressed with CH and 1NH under P*nah* in strain CSV86^T^ referred to as CSV86-MEC2, which showed improved growth on Carbaryl compared with CSV86-MEC1 (30). The choice of P*nah* promoter was crucial as its leaky expression allowed basal levels of McbT, CH, and 1NH, sufficient enough to initiate Carbaryl uptake and initial metabolism. As salicylate accumulated intracellularly, it activated the endogenous NahR regulator, thereby inducing the native *nah* and *sal* operons for downstream metabolism (Fig. 5B). This self-amplifying regulatory loop tightly integrated the recombinant modules from strain C5pp with the native metabolic network of strain CSV86^T^, ensuring complete mineralization of Carbaryl. Moreover, Carbaryl was preferentially utilized over glucose and co-metabolized with organic acid succinate.

## OUTLOOK AND WAY AHEAD

**Fig 6:**
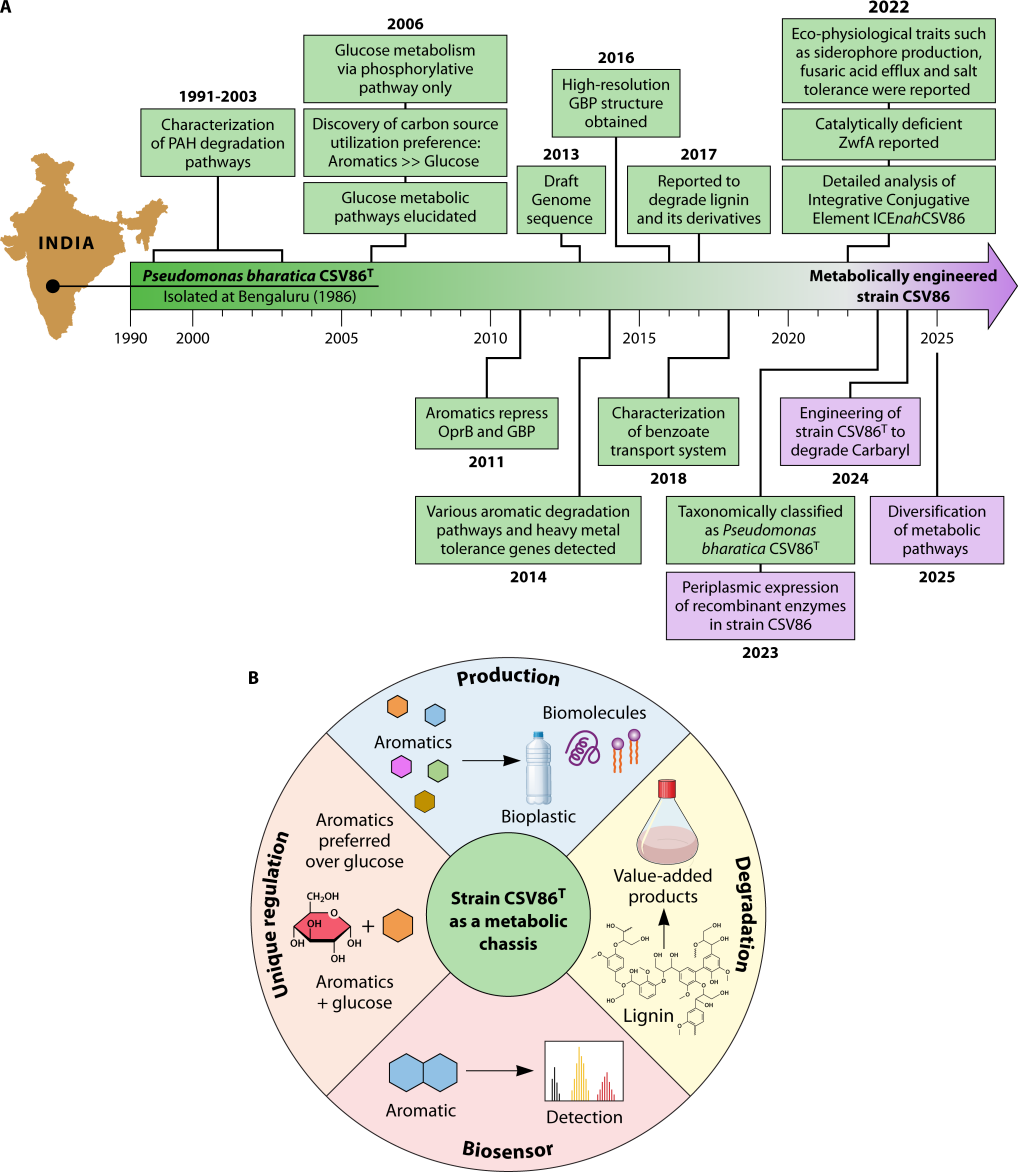
Progression of major research findings and future applications of *Pseudomonas bharatica* CSV86^T^. (**A**) Timeline of key research progress. (**B**) Prospective applications of strain CSV86^T^ for detection, degradation, and up-cycling of aromatics.

Both wild-type and engineered *Pseudomonas* strains represent promising platforms for bioconversion and biotransformation toward the sustainable synthesis of value-added products. These capabilities are enabled through *de novo* biosynthesis from renewable feedstocks, biotransformation in fermentation systems, metabolic funneling of lignin-derived phenylpropanoids, and the upcycling of aromatic monomers from waste, such as plastic (95, 96). In the past 25 years, comprehensive molecular and biochemical studies of aromatic-degrading soil bacterium, *Pseudomonas bharatica* CSV86^T^, revealed the metabolic pathways, genes, key enzymes, carbon source utilization hierarchy, and various eco-physiological traits, highlighting its metabolic flexibility, plasticity, and ecological adaptation (Fig. 6A). We are better poised to move beyond understanding “what and how it does it“ toward engineering it for “what we want it to do.” The goal is to develop strain CSV86^T^ as a customizable platform for programmable detection, efficient degradation, and sustainable up-cycling of a wide variety of aromatic compounds (Fig. 6B).

One of the most unique features of strain CSV86^T^ is preferential utilization of aromatic compounds over simple carbon sources, which makes it a superior candidate for biodegradation of aromatics in the environment, i.e., bioremediation process (6, 14). Multi-OMICS coupled with systems biology tools presents as promising approaches to systematically explore the regulatory nodes controlling carbon flux, transcriptional regulation, and CCR bypass mechanisms (39). It has implications in rewiring CCR in related bacteria for targeted metabolic functions under mixed-substrate conditions. The unique eco-physiological traits of strain CSV86^T^ make it a promising candidate for development as a metabolic chassis. Development of genome-scale metabolic models and genome reduction/streamlining will aid in designing efficient engineering strategies and improve its robustness and productivity (97–100). With diverse pathways for aromatic substrates and robust metabolic network, strain CSV86^T^ presents immense potential to be used as a microbial cell factory for up-cycling aromatic pollutants into high-value products like bioplastics and pharmaceutical precursors.

One of the challenges in metabolic engineering for producing bio-valued products is reducing metabolic crosstalk. Compartmentalization engineering is an efficient strategy to limit crosstalk between the engineered pathway and the cellular milieu (101). Periplasmic localization of enzymes/pathways could be employed as an alternate strategy (101–103). Moreover, periplasmic localization simplifies the purification of recombinant proteins as described for recombinant CH enzyme from the periplasmic space of strain CSV86^T^ by single-step affinity chromatography (90). As discussed earlier, transmembrane domain and signal peptide (Tmd+Sp) together facilitate the translocation of mature CH to the periplasmic space in strain CSV86^T^. Thus, Tmd+Sp can be employed to translocate industrially relevant enzymes to the periplasmic space for the production of bio-valued products or mineralization of toxic pollutants in strain CSV86^T^. Downstream processing and extraction of metabolites from the periplasmic space are relatively easy compared with the cytoplasm. In addition, expression and localization of McbT to the outer membrane of CSV86^T^ also demonstrate the utility of the signal peptide of McbT to localize proteins into the outer membrane of CSV86^T^. Also, exploring cell surface display and/or extracellular secretion of enzymes in strain CSV86^T^ could offer further advantage for its application at industrial scale.

The ability to bypass CCR makes strain CSV86^T^ a desirable host for construction of whole cell biosensors for detection of aromatic pollutants in complex samples containing simple carbon sources. While laboratory-scale proof-of-concept studies hold potential, translating these concepts into real-world applications requires strain optimization and ecological testing of CSV86^T^ at large scale in pilot fields or industrial settings to evaluate survival, activity, pollutant degradation efficiency, and ecological impact. Ensuring biosafety and developing regulatory frameworks for the use of engineered strain CSV86^T^ will be crucial towards practical applications.
